# Real world analysis of high-cut-off (HCO) hemodialysis with bortezomib-based backbone therapy in patients with multiple myeloma and acute kidney injury

**DOI:** 10.1007/s40620-020-00939-2

**Published:** 2020-12-31

**Authors:** N. Steiner, A. Abdel Hamid, A. Kronbichler, H. Neuwirt, M. Myslivecek, M. Kollar, J. Lachmanova, R. Rysava, Z. Hruskova, I. Spicka, W. Willenbacher, D. Nachbaur, D. Wolf, V. Tesar, E. Gunsilius

**Affiliations:** 1 Department of Internal Medicine V, Hematology and Oncology, Medical University of Innsbruck, Anichstr. 35, 6020 Innsbruck, Austria; 2 Department of Internal Medicine IV, Nephrology and Hypertension, Medical University of Innsbruck, Innsbruck, Austria; 3 Department of Nephrology, First Faculty of Medicine, Charles University and General University Hospital in Prague, Prague, Czech Republic; 4 Department of Clinical and Transplant Pathology, Institute for Clinical and Experimental Medicine, Prague, Czech Republic; 5 First Department of Internal Medicine and Hematology, First Faculty of Medicine, Charles University and General University Hospital in Prague, Prague, Czech Republic

**Keywords:** High-cut-off (HCO) hemodialysis, Multiple myeloma, Bortezomib, Cast nephropathy, Acute kidney injury

## Abstract

**Background:**

In patients with multiple myeloma (MM) free light chain-induced cast nephropathy is a serious complication associated with poor survival. High-cut-off (HCO) hemodialysis can reduce the amount of serum free light chains (sFLC), but data on its impact on clinical outcome is limited and contradictory. To gain further insights we collected real world data from two major myeloma and nephrology centers in Austria and the Czech Republic.

**Methods:**

Sixty-one patients with MM and acute kidney injury, who were treated between 2011 and 2019 with HCO hemodialysis and bortezomib-based MM therapy, were analyzed.

**Results:**

The median number of HCO hemodialysis sessions was 11 (range 1–42). Median glomerular filtration rate at diagnosis was 7 ± 4.2 ml/min/1.73m^2^. sFLC after the first HCO hemodialysis decreased by 66.5% and by 89.2% at day 18. At 3 and 6 months, 26 (42.6%) and 30 (49.2%) of patients became dialysis-independent.

**Conclusion:**

The widely used strategy combining HCO hemodialysis and bortezomib-based antimyeloma treatment is dissatisfactory for half of the patients undergoing it and clearly in need of improvement.

**Supplementary Information:**

The online version contains supplementary material available at 10.1007/s40620-020-00939-2.

## Introduction

Patients with multiple myeloma (MM) frequently present with acute kidney injury (AKI) at the time of diagnosis. Since light chains are filtered through the glomeruli, the increased production of free light chains (FLCs) exceeds the resorption capacity of the proximal tubules. Precipitation occurs at the Tamm-Horsfall protein (uromodulin) in the distal tubule, thus forming insoluble aggregates and casts that may eventually lead to kidney failure [1]. In patients with MM, FLC-induced cast nephropathy is a serious complication associated with poorer survival.

In a recent population-based study of 1038 newly diagnosed myeloma patients, 25% presented with any degree of kidney failure and 13% required dialysis. Median survival was only 21 months in patients with kidney failure, a value that other patients did not achieve after three years (P < 0.01). Factors associated with 1-year overall survival were myeloma [hazard ratio (HR) 0.13; P < 0.01] and response in terms of improving kidney function (HR 0.27; P = 0.03). Thus, recovery of kidney function is one of the strongest parameters for survival [2].

In addition to the immediate application of anti-myeloma agents to reduce the production of serum free light chains (sFLCs), the reduction of high sFLC concentrations can be achieved by extracorporeal techniques. High cut-off (HCO) dialysis has been used in cast nephropathy due to MM. It combines the kidney replacement therapy required due to kidney failure and the elimination of FLCs. In HCO dialysis, dialyzers with a high permeability for molecules (up to 50 kDa) are used. This special filter enables the removal of large amounts of FLCs in the serum and thus effectively lowers their concentration. In this way, kidney failure may be reversed [3]. HCO dialysis is performed in conjunction with systemic therapy to minimize the production of FLCs. The proteasome inhibitor bortezomib is recommended as it is also effective in patients with kidney function impairment and can be used without primary dose reduction. However, clinical trials and data on the additional benefit of HCO hemodialysis (HD) in combination with novel therapeutic agents like bortezomib are lacking and, as a consequence of two randomized studies, the use of HCO-HD is discussed controversially [4–6]. The aim of this retrospective study was to investigate the efficacy of combined multi-modality treatment in a real-life setting, and to add these data to the existing literature to conceptualize optimized treatment modalities for these patients.

## Methods

The analysis included 61 patients with MM and AKI, who were treated between 2011 and 2019 with HCO dialysis and bortezomib-based chemotherapy either at the Department of Hematology and Oncology of the Medical University of Innsbruck (23 patients) or at the Department of Nephrology and 1st Internal Clinic-Hematology-of the General University Hospital in Prague (38 patients). All patients presented with severe AKI requiring dialysis. Approval for data collection and publication was obtained from the Ethics Committee of the Medical University of Innsbruck (vote #1045/2019). Serum FLC concentrations were measured with the FREELITE™ immunoassay immediately before and after each dialysis session after initiation of HCO dialysis. Glomerular filtration rate was calculated using the CKD-EPI equation. The primary outcome was recovery of kidney function (defined as independence from dialysis) at 3 months from diagnosis. The secondary endpoint was independence from dialysis at 6 months from diagnosis. Mortality during the HCO-HD) treatment, at three and six months was also assessed. Outcome of kidney function was evaluated with regard to myeloma response in a subset of 31 patients.

## Results

### Patient characteristics

The final dataset consisted of 61 patients with newly diagnosed MM and dialysis-dependent AKI. The patients’ demographics, clinical characteristics and treatment details are shown in Table 1. Median age of the patients was 66 (range 37–91) years; 82% had MM ISS Stage III and 11.5% had Stage II. One patient (1.6%) had ISS Stage I. Thirty-four (55.7%) patients had light-chain myeloma, while 27 (44.3%) had complete clonal immunoglobulins. Patients with ISS Stage II, ISS Stage III, or the genetic aberrations del17p, t(4;14), amp1q or hypodiploid karyotypes were classified as high-risk patients. Diagnosis of cast nephropathy was confirmed by kidney biopsy including immunofluorescence, light microscopy and electron microscopy in 12 patients (19.7%). Light chain deposition disease was detected in one patient with cast nephropathy (8%, 1/12). Other diseases usually associated with MM, such as AL amyloidosis or light chain proximal tubulopathy were not confirmed in kidney biopsies. Vascular changes including vascular nephrosclerosis and hypertensive microangiopathy were present in 7 patients (58%, 7/12) and vascular pathology with diabetic kidney disease in 2 patients (17%, 2/12). For more detailed analysis of kidney biopsy findings see Supplementary Table 1. All patients underwent HCO dialysis and were additionally treated with bortezomib-based therapy. 54 (88.5%) patients received bortezomib, an immunomodulator and dexamethasone, while seven (11.5%) received bortezomib and dexamethasone (Table 2).

**Table 1:** Patient demographics and clinical characteristics

Parameter	N = 61	%
Center		
Prague	38	62.3
Innsbruck	23	37.7
Sex		
Female	19	68.9
Male	42	31.1
Age		
Median age (range), years	66 (37–91)	
< 65 years	25	41
≥ 65 years	36	59
Isotype		
Heavy and light chain	27	44.3
Only light chain	34	55.7
ISS Stage		
Stage I	1	1.6
Stage II	7	11.5
Stage III	50	82
Missing	3	4.9
High-risk patients (classified as patients with ISS Stage II or III, del17p, t(4;14), amp1q or hypodiploid karyotype)		
High	57	93.5
Low	1	1.6
Missing	3	4.9
CRAB (hypercalcemia, renal insufficiency, anemia, bone disease) criteria		
1 CRAB criteria only	6	9.8
2 CRAB criteria	18	29.5
3 CRAB criteria	20	32.7
4 CRAB criteria	17	27.9

**Table 2:** Kidney-related and myeloma parameters before commencement of HCO-HD

	N = 61	%
Conventional HD before commencement of HCO-HD		
Yes	27	44.3
No	34	55.7
Serum creatinine before commencement of HCO-HD if not already on HD		mg/dl
Mean		6.2
Median		6.0 ± 2.4
Minimum		2.7
Maximum		11.5
Percentile 25		4.7
Percentile 75		7.2
Cast nephropathy confirmed by renal biopsy	N = 61	%
Yes	12	19.7
No	49	80.3
Serum FLC levels before commencement of HCO-HD		mg/l
Mean		11,662.5
Median		7883 ± 13,464.2
Minimum		334
Maximum		86,613
Percentile 25		4627
Percentile 75		13,325

### Kidney function at diagnosis

Of the study patients 27 (44.3%) underwent HD treatment prior to HCO dialysis. Median glomerular filtration rate at time of diagnosis of patients without HD prior to HCO dialysis was 7 ml/min/1.73m^2^ with a standard deviation of ± 4.2 ml/min/1.73m^2^. Patients received HCO dialysis for a median of 11 days, with a minimum HCO dialysis duration of one day and a maximum HCO dialysis duration of 42 days.

### Serum FLC during HCO-HD

**Fig. 1:**
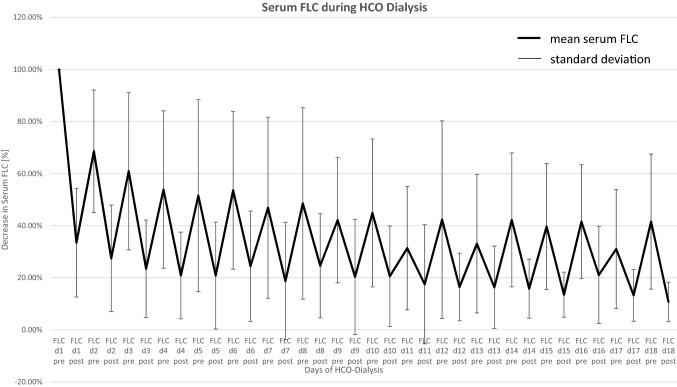
sFLC decline and rebound after and before each HCO-HD session

The course of FLCs before and after each HCO-HD is shown in Fig. 1. In brief, the concentration of sFLC after the first HCO dialysis decreased by 66.5% (SD ± 20.88%) with a 35% rebound before the next HCO-HD. On the 18th HCO dialysis day, the sFLC concentration was 10.8% of the initial concentration (SD ± 7.52%). Thus, a reduction of 89.2% from baseline was achieved.

### Kidney function and the rate of kidney recovery (off dialysis) at three- and six-months follow-up

Tables 3 and 4 show the overall kidney recovery rate, defined as dialysis independency at 3 months and 6 months after the last HCO dialysis, which was 42.6% (26 patients) and 49.2% (30 patients), respectively. Patients achieving dialysis independency at 3 and 6 months had a median serum creatinine of 1.3 ± 1.3 mg/dl (3 months follow-up) and 1.5 ± 1.4 mg/dl (6 months follow-up), respectively (Table 4). Four (6.6%) patients died during therapy or up to 4 weeks after the last HCO dialysis due to progression of MM (Table 3). HCO-HD-associated complications were not observed (Table 5).

**Table 3:** Mortality on HCO-HD treatment and during follow-up

	N = 61	%
Alive at discontinuation of HCO-HD treatment		
Yes	57	93.4
No	4	6.6
Alive at 3 months		
Yes	53	86.9
No	8	13.1
Alive at 6 months		
Yes	49	80.3
No	12	19.7

**Table 4:** Kidney function parameters follow-up at 3 and 6 months

	N = 53	%
Renal recovery at 3 months (dialysis-free)		
Yes	26	49.1
No	27	50.9
Renal recovery at 6 months (dialysis-free)	N = 49	%
Yes	30	61.2
No	19	38.8
Creatinine at 3 months if dialysis-free (n = 21)		mg/dl
Mean		1.74
Median		1.3 ± 1.3
Minimum		0.7
Maximum		4.7
Percentile 25		0.8
Percentile 75		2.1
Creatinine at 6 months if dialysis-free (n = 24)		mg/dl
Mean		2
Median		1.5 ± 1.4
Minimum		0.6
Maximum		5.1
Percentile 25		0.9
Percentile 75		3.0

**Table 5:** HCO-HD treatment and FLC outcomes

Number of HCO-HDs per patient	
Mean	13.2
Median	11 ± 9.2
Minimum	1
Maximum	42
Percentile 25	7
Percentile 75	16
Serum FLC levels before the last HCO-HD	mg/l
Mean	2315.2
Median	1209.5 ± 3067.1
Minimum	30
Maximum	14,335
Percentile 25	521.3
Percentile 75	2656
Average reduction in serum FLCs per one HCO-HD session	%
Mean	65.8
Median	68.8 ± 17.2
Minimum	17.7
Maximum	97.6
Percentile 25	61.9
Percentile 75	77.2

### Myeloma response

Myeloma response after the first line of chemotherapy was observed in most of the patients from Prague (n = 33) and is shown in Table 6. Of the 18 patients who achieved complete remission (CR) or very good partial response (VGPR), 13 patients were HD-independent at three months and 17 patients at six months (Tables 6 and 7).

**Table 6:** Myeloma response after the first line of chemotherapy

	N = 61	%
Hematological response based on Multiple Myeloma Response Criteria		
sCR	1	1.64
CR	5	8.2
VGPR	12	19.67
PR	7	11.48
SD	1	1.64
PD	5	8.2
Died during treatment period	1	1.64
Not known	28	45.9

*sCR* stringent complete remission, *CR* complete remission, *VGPR* very good partial response, *PR* partial response, *MR* minimal response, *SD* stable disease, *PD* progressive disease

**Table 7:** Outcomes per myeloma response

Mortality per hematological response	N = 31	Alive at 3 months	Alive at 6 months
sCR or CR	6	6	6
VGPR	12	12	12
PR	7	7	5
SD or PD	6	6	5

Renal outcomes per hematological response	N = 28	Off dialysis at 3 months	Off dialysis at 6 months
sCR or CR	6	5	5
VGPR	12	8	12
PR	5	3	3
SD or PD	5	2	2

### Subgroup analysis based on the kidney recovery rate

Twenty-six patients were HD-independent at three months, while 27 were HD-dependent. sFLC levels at the beginning of HCO-HD treatment were similar in both groups. However, sFLCs at cessation of HCO-HD (measured before the last HCO-HD) tended to be higher in those patients who remained HD-dependent despite having had a larger number of HCO-HD sessions, although this difference was not statistically significant (Table 8). Similar results were also noted at six months. This suggests that these patients do not respond well to bortezomib-based antimyeloma treatment and produce excessive amounts of FLCs despite their extracorporeal removal.

**Table 8:** Subgroup analysis per kidney outcome

Renal outcome at 3 months (n = 53)	Dialysis free n = 26	Dialysis-dependent n = 27
Number of HCO-HDs		
Mean	11.6	14.5
Median	11 ± 6	13 ± 11
Minimum	3	1
Maximum	27	42
Percentile 25	7	5.5
Percentile 75	14.3	23.3
FLC at commencement of HCO-HD	mg/l	mg/l
Mean	8089	14,565
Median	6304 ± 6993	8440 ± 16,516
Minimum	334	1111
Maximum	27,205	86,613
Percentile 25	2530	5646
Percentile 75	12,700	13,850
FLC before the last HCO-HD	mg/l	mg/l
Mean	2313	2382
Median	1128 ± 3331	1607 ± 2937
Minimum	30	64
Maximum	14,335	13,574
Percentile 25	573	514
Percentile 75	2537	2844

Renal outcome at 6 months (n = 49)	Dialysis-free n = 30	Dialysis-dependent n = 19
Number of HCO-HDs		
Mean	11	15.2
Median	11 ± 6	13.5 ± 11.3
Minimum	2	1
Maximum	27	42
Percentile 25	7	6.8
Percentile 75	13.3	24.5
FLC at commencement of HCO-HD	mg/l	mg/l
Mean	8612	15,028
Median	7460 ± 6700	8038 ± 17,485
Minimum	334	1320
Maximum	27,205	86,613
Percentile 25	2670	5646
Percentile 75	12,806	16,745
FLC before the last HCO-HD	mg/l	mg/l
Mean	2175	2463
Median	1128 ± 3121	1607 ± 3115
Minimum	30	112
Maximum	14,335	13,574
Percentile 25	573	497
Percentile 75	2538	3412

## Discussion

Impairment of kidney function is an independent negative prognostic factor of capital importance in MM. Recovery of kidney function and hematologic response are the strongest markers associated with patient survival [2]. There is a relationship between the rapid reduction of FLCs in serum and the improvement of kidney function [7, 8]. In a large cohort study of 1542 patients, kidney impairment was one of the major causes of early death after diagnosis of MM [9]. Since the development of AKI predicts poor prognosis for MM patients and the cast nephropathy-induced AKI is due to elevated FLC production by the plasma cells, the most important predictive factor for kidney recovery is rapid reduction of sFLC either by removing them from serum or by inhibiting their production with potent anti-myeloma drugs [10, 11]. The role of novel anti-myeloma agents, i.e. proteasome inhibitors such as bortezomib in the management of patients with MM presenting with severe kidney impairment was evaluated in the EULITE study [5]. After 90 days, 24 (56%) patients in the HCO-HD group and 24 (51%) in the high-flux hemodialysis (HF-HD) group were independent of dialysis (P = 0.81). In the MYRE study [4], dialysis independency was achieved in 33% and 43% of study patients at three months and in 37.5% and 60% at six months in the control and HCO arms, respectively. The removal of FLCs via HCO dialysis without appropriate chemotherapy has only a minor effect due to the high rate of FLC production in patients with untreated MM and the rapid re-diffusion from tissues into the bloodstream [12]. Especially the use of the proteasome inhibitor bortezomib seems to be beneficial as it requires no dose-adjustment in patients with kidney impairment [13, 14]. Thus, in many centers a bortezomib‐containing regimen is actually considered the treatment of choice for MM patients presenting with AKI.

Recently, studies using HCO-HD have shown both, in vitro and in vivo, a high efficacy for sFLC removal [15]. In our study, at the individual end of HCO-HD treatment, the mean FLC serum concentration was 10.8% from baseline, which corresponds to a reduction of 89.2%. Our study shows a considerable sFLC clearance with HCO-HD combined with bortezomib treatment [6, 16]. Also, the median glomerular filtration rate increased substantially and clinically meaningfully, i.e. half of the patients became dialysis-independent. These data are in line with those of Hutchison et al. [12], who reported a kidney recovery rate of 65% at month 3 of treatment with HCO-HD and bortezomib, and an estimated glomerular filtration rate of 40 (range 11–83) ml/min/1.73m^2^. While a randomized controlled trial (59 patients) showed a dialysis independence rate of 41% for the HCO dialysis therapy group, a retrospective case–control study (98 patients) reported a 65.6% rate of dialysis independency [16, 17]. Nevertheless, the randomized EULITE trial showed no overall benefit regarding dialysis independence for patients on HCO dialysis as compared to HF-HD [5]. The MYRE trial also did not meet the primary outcome defined as dialysis independence at three months, but met the secondary outcome at six months [4].

Our data come from a real-world analysis of patients with newly diagnosed MM complicated by AKI requiring kidney replacement therapy. As per the current recommendations, HCO dialysis was administered to these patients if decided by the treating physician. The kidney recovery rate in our patients was 42.6% at three months and 49.2% at 6 months, which is similar to previously published data in the era of novel anti-myeloma drugs. It also suggests that kidney recovery can take longer than three months in the case of cast nephropathy. Patients who achieved complete remission or very good partial response after the first line of chemotherapy had, as anticipated, a better prognosis in terms of mortality, but also in terms of kidney outcome.

In summary, facilitated removal of sFLCs by extracorporeal techniques did not show a significant benefit in terms of kidney recovery. Both randomized trials had their limitations in terms of sample size and selection bias [18].

Our study was not interventional and thus no firm conclusions can be drawn from our results. It should also be noted that a large heterogeneity was present in our cohort, which is another limitation of the current work. However, it seems that patients in whom the antimyeloma treatment is not successful do not benefit from HCO-HD treatment, as the rapid production and re-distribution of FLCs prevent sufficient elimination by facilitated removal.

We confirm that HCO-HD combined with bortezomib treatment is effective, but still dissatisfactory and we propose a strategy for rapid and profound suppression of light chain production by complementary administration of the most potent anti-myeloma compounds in combination with conventional non-HCO-HD.

Unfortunately, myeloma patients with severe kidney impairment are typically excluded from clinical trials testing modern anti-myeloma compounds. To corroborate the proposed strategy, we are currently running a phase 2 trial designed exclusively for such patients, where a modern quadruplet combination therapy accompanied by conventional dialysis is administered.

## Supplementary Information

Below is the link to the electronic supplementary material.

Supplementary file1 (DOCX 14 KB)

## References

[1] Dimopoulos MA, Kastritis E, Rosinol L, Bladé J, Ludwig H (2008). Pathogenesis and treatment of renal failure in multiple myeloma. Leukemia.

[2] Courant M, Orazio S, Monnereau A, Preterre J, Combe C, Rigothier C (2019) Incidence , prognostic impact and clinical outcomes of renal impairment in patients with multiple myeloma : a population-based registry. Nephrol Dial Transplant 1–9 10.1093/ndt/gfz211 31773154

[3] Hutchison CA, Heyne N, Airia P, Schindler R, Zickler D, Cook M (2012). Immunoglobulin free light chain levels and recovery from myeloma kidney on treatment with chemotherapy and high cut-off haemodialysis. Nephrol Dial Transplant.

[4] Bridoux F, Pegourie B, Augeul-Meunier K, Royer B, Joly B, Lamy T (2016). Treatment of myeloma cast nephropathy (MCN): a randomized trial comparing intensive haemodialysis (HD) with high cut-off (HCO) or standard high-flux dialyzer in patients receiving a bortezomib-based regimen (the MYRE Study, by the Intergroupe Francophone. Blood.

[5] Hutchison CA, Cockwell P, Moroz V, Bradwell AR, Fifer L, Gillmore JD (2019). High cutoff versus high-flux haemodialysis for myeloma cast nephropathy in patients receiving bortezomib-based chemotherapy (EuLITE): a phase 2 randomised controlled trial. Lancet Haematol.

[6] Cook M, Hutchison C, Fifer L, Gillmore J, Heyne N, Weisel K et al. (2016) High cut-off haemodialysis (hco-hd) does not improve outcomes in myeloma cast nephropathy: results of european trial of free light chain removal by extended haemodialysis in cast nephropathy (EuLITE) [poster presentation]. European Hematology Association (EHA) Learning Center, The Hague, The Netherlands

[7] Hutchison CA, Cockwell P, Stringer S, Bradwell A, Cook M, Gertz MA (2011). Early reduction of serum-free light chains associates with renal recovery in myeloma kidney. J Am Soc Nephrol.

[8] Leung N, Gertz MA, Zeldenrust SR, Rajkumar SV, Dispenzieri A, Fervenza FC (2008). Improvement of cast nephropathy with plasma exchange depends on the diagnosis and on reduction of serum free light chains. Kidney Int.

[9] Uttervall K, Andreasson J, Liwing J, Näsman P, Aschan J, Nahi H (2012). Is renal impairment still a poor prognostic marker in myeloma care? A population based study including 1542 patients. Am Soc Hematol.

[10] Dimopoulos MA, Delimpasi S, Katodritou E, Vassou A, Kyrtsonis MC, Repousis P (2014). Significant improvement in the survival of patients with multiple myeloma presenting with severe renal impairment after the introduction of novel agents. Ann Oncol.

[11] Cockwell P, Hutchison CA (2010) Management options for cast nephropathy in multiple myeloma. Curr Opin Nephrol Hypertens [Internet].19(6). Available from: https://journals.lww.com/co-nephrolhypertens/Fulltext/2010/11000/Management_options_for_cast_nephropathy_in.7.aspx 10.1097/MNH.0b013e32833ef72c 20827195

[12] Hutchison CA, Bradwell AR, Cook M, Basnayake K, Basu S, Harding S (2009). Treatment of acute renal failure secondary to multiple myeloma with chemotherapy and extended high cut-off hemodialysis. Clin J Am Soc Nephrol.

[13] Ludwig H, Adam Z, Hajek R, Greil R, Tóthová E, Keil F (2010). Light chain-induced acute renal failure can be reversed by bortezomib-doxorubicin-dexamethasone in multiple myeloma: results of a phase II study. J Clin Oncol.

[14] Dimopoulos MA, Roussou M, Gavriatopoulou M, Zagouri F, Migkou M, Matsouka C (2009). Reversibility of renal impairment in patients with multiple myeloma treated with bortezomib-based regimens: identification of predictive factors. Clin Lymphoma Myeloma.

[15] Hutchison CA, Cockwell P, Reid S, Chandler K, Mead GP, Harrison J (2007). Efficient removal of immunoglobulin free light chains by hemodialysis for multiple myeloma: in vitro and in vivo studies. J Am Soc Nephrol.

[16] Gerth HU, Pohlen M, Görlich D, Thölking G, Kropff M, Berdel WE (2016). Impact of high-cut-off dialysis on renal recovery in dialysis-dependent multiple myeloma patients: Results from a case-control study. PLoS ONE.

[17] Bridoux F, Carron P-L, Pegourie B, Alamartine E, Augeul-Meunier K, Karras A (2017). Effect of high-cutoff hemodialysis vs conventional hemodialysis on hemodialysis independence among patients with myeloma cast nephropathy: a randomized clinical trial. JAMA.

[18] Bridoux F, Chevret S, Fermand JP (2019). High cutoff haemodialysis in myeloma cast nephropathy: further investigation is needed. Lancet Haematol.

