# Supplementary material for: Real world analysis of high-cut-off (HCO) hemodialysis with bortezomib-based backbone therapy in patients with multiple myeloma and acute kidney injury
Source: J Nephrol. 2020 Dec 31;34(4):1263–70. doi: 10.1007/s40620-020-00939-2 (PMC8357738; doi:10.1007/s40620-020-00939-2)
Supplement: Supplementary file 1 — Supplementary file1 (DOCX 14 KB) [file 40620_2020_939_MOESM1_ESM.docx]

| **Patient no.** | **Age/ Gender** | **Dialysis at dg (Y/N)** | **Kidney biopsy findings** | **Alive at 3m (Y/N) Alive at 6m (Y/N)** | **Off dialysis at 3m (Y/N) Off dialysis at 6m (Y/N)** |
| --- | --- | --- | --- | --- | --- |
| 1 | 60/ F | N | LCCN | Y/Y | N/Y |
| 2 | 49/ M | Y | LCCN, LCDD and hypertensive changes | Y/Y | N/Y |
| 3 | 64/ F | N | LCCN | Y/Y | Y/Y |
| 4 | 76/ M | N | LCCN and diabetic kidney disease | Y/Y | Y/Y |
| 5 | 75/ F | Y | LCCN, diabetic kidney disease and hypertensive changes | Y/Y | Y/Y |
| 6 | 77/ F | Y | LCCN and hypertensive changes | Y/Y | N/N |
| 7 | 69/ M | N | LCCN | Y/Y | Y/Y |
| 8 | 43/ M | N | LCCN and hypertensive changes | Y/Y | Y/Y |
| 9 | 69/ M | N | LCCN | Y/Y | Y/Y |
| 10 | 67/F | N | LCCN and hypertensive changes | Y/Y | Y/Y |
| 11 | 65/ M | Y | LCCN and hypertensive changes | Y/N | N/ - |
| 12 | 52/ F | Y | LCCN and hypertensive changes | Y/Y | N/Y |

Supplementary Table 1: Characteristics of the 12 patients undergoing kidney biopsy before initiation of HCO-dialysis.

Abbreviations used: F (female), M (male), LCCN (light chain cast nephropathy), LCDD (light chain deposition disease)

**Histopathology of cast nephropathy:**

The casts were usually strongly positive for the patients’s dominant light chain in immunofluorescence. Light chain casts were typically localized in tubular lumina and appeared fractured with angulated edges with rectangular or rhomboid shape. These intratubular casts were associated with a cellular reaction including neutrophils and focally also giant cells. The casts were distinguished from other tubular protein material by negative or weak positive Periodic Acid Shiff (PAS). Intraluminal presence of light chain casts, sporadically also with epithelial damage caused acute tubular injury, the correlation of AKI. Edema or sparse fibrosis was present in interstitium, which contains an inflammatory infiltrate composed of lymphocytes, focally admixed with neutrophils and eosinophils.
